# Experimental and Numerical Study of Surface Roughness of Thin Brass Wire Processed by Different Dieless Drawing Processes

**DOI:** 10.3390/ma15010035

**Published:** 2021-12-21

**Authors:** Andrij Milenin, Mirosław Wróbel, Piotr Kustra, Jiří Němeček

**Affiliations:** 1 Faculty of Metals Engineering and Industrial Computer Science, AGH University of Science and Technology, 30-059 Cracow, Poland; milenin@agh.edu.pl (A.M.); mwrobel@agh.edu.pl (M.W.); 2 Faculty of Civil Engineering, Czech Technical University in Prague, 160 00 Prague, Czech Republic; jiri.nemecek@fsv.cvut.cz

**Keywords:** dieless drawing, wires, brass, roughness

## Abstract

This paper examines the surface roughness of a thin brass wire (140–200 microns in diameter) after two dieless drawing (DD) processes, i.e., conventional dieless drawing (CDD) and incremental dieless drawing (IDD). In incremental dieless drawing, small increments in deformation were applied in several passes. It has been proven that the IDD process not only has a greater efficiency but also enables obtaining a wire with significantly lower surface roughness. The explanation for these effects is based on the results of the numerical modeling of both compared processes. The developed numerical model takes into consideration the initial roughness of the wire surface, shape and dimensions of grains, and their diversified mechanical properties. Nanoindentation measurements, microstructure, and plastometric studies allowed us to find the effective flow stress distribution in the grains. The IDD process was found to be much more stable and develop a much more uniform distribution of grain strain than the CDD process. More homogeneous deformation results in surface roughness reduction. Approximately 25–30% reduction in surface roughness of the wire produced by the IDD process was predicted by simulations and confirmed experimentally.

## 1. Introduction

The dieless drawing (DD) process consists of stretching a workpiece that has been locally heated in such a way that the plastic deformation is localized in the material volume with increased temperature. The workpiece movement relative to the heating device ensures the processing of long products (e.g., wires). To the best of our knowledge, this process was proposed for the first time by Weiss and Kot for wire production [1]. Currently, this process is laboratory tested for the manufacture of pipes [2], bars [3], and wires [4]. The lack of dies and lubricants enables significant cost reduction [5] and eliminates the influence of friction on the deformation process [6]. These are the significant advantages of this technology. Moreover, the appropriate selection of deformation temperature and strain rate enables the processing of materials with limited plasticity [7]. On the other hand, geometrically unconstrained deformation can result in product diameter heterogeneity along the length [8]. There are two methods to obtain it, which can both be used independently and simultaneously. The first method is to control the cooling speed of the processed material as it extends from the deformation zone [9]. This method can also be used to obtain a product with a prescribed length-varying diameter [10]. The effectiveness of this method depends on the material flow stress sensitivity to the deformation temperature. The second method is to use a multi-pass deformation process, proposed for the first time by Furushima and Manabe [11]. The maximum single-pass deformation that did not cause product diameter heterogeneity was chosen by them as the crucial process parameter. This approach is quite practical, but it is not based on any theoretical explanation. Such an explanation was proposed by Milenin [12], and this approach was used in the study presented in this paper. Thus, the idea developed in this work is that the DD process is divided into several passes with a small increment of strain in each of them. For each of the strain increments, stability of the plastic deformation was determined based on the Considère criterion ([13], as well as some 21st-century literature, e.g., [14,15]) proposed by Byla et al. [16]:

(1) $$K_{stab}=\frac{d\sigma }{d\varepsilon }\frac{1}{\sigma }$$

where *ε* is the strain, and σ is the stress determined from the flow stress–strain curve of the deformed material. Value *K_stab_* < 1 means non-stable plastic deformation, and *K_stab_* > 1 means stable deformation.

The stability of the plastic deformation increases with the value of *K_stab_*. At the same time, the tendency to localize the deformation in the form of necking decreases, which has been confirmed experimentally previously (e.g., [17]). Knowledge of the *K_stab_* value allows for precise control of the necking through precise selection of the deformation rate. Such a methodology (known as incremental dieless drawing (IDD)) requires knowledge of the flow stress dependence on the strain, strain rate, and temperature. Previously, this methodology was successfully applied to improve the efficiency of the DD process and to prove that the workability of the IDD process is significantly higher than that of the conventional DD process (CDD) [17].

According to Equation (1), the stability of plastic deformation (coefficient *K_stab_*) increases with a decreasing strain increment. Usually, it is necessary to recover the deformed material ductility between some increments in deformation. During dieless drawing, this is usually guaranteed by the high deformation temperature at which recrystallization occurs [12,17]. On the other hand, too-small strain increments significantly reduce the productivity of the process, since it requires multiple repetitions (i.e., multi-pass process). Thus, it is most rational to determine the optimal value of the strain increment based on an experiment.

In this study, the IDD process was used both to improve the workability and to reduce the product surface roughness.

Minimizing roughness is especially important for very fine wires. In general, roughness can be roughly regarded as the geometrical heterogeneity of the product on the microscale. Minimizing roughness is important for at least two reasons. First, an increase in roughness can be the onset of tensile necking. Second, surface roughness can reduce the mechanical, corrosive, and electrical performance of the wire. An increase in surface roughness during deformation is well known as strain-induced roughness. It has been confirmed for various materials, e.g., steel [18], aluminum alloys [19], and titanium [20]. Strain-induced roughness was investigated by Dai and Chiang [21]. They concluded that roughness is proportional to the amount of imposed plastic strain and the average grain size. A monotonic increase in roughness as a function of the initial roughness and the strain imposed at room temperature was proven experimentally by Stoudt et al. [22]. On the other hand, reduction in roughness due to multi-pass DD was reported by Furushima and Manabe [11]. An attempt was made to explain this effect as a result of surface profile elongation with drawing wire elongation [23]. However, this explanation does not answer the question of why this effect is much more pronounced for multi-pass IDD than for single-pass DD with the same total deformation. Answering this question is one of the goals of this paper. Experimental studies are insufficient to explain this. First, the process of roughness measurement is accompanied by sufficiently large errors, which can be comparable with the magnitude of the effect under study. Second, it is difficult to understand the mechanism of the observed phenomenon only based on an experiment. For these reasons, in this paper, along with experimental studies, numerical simulation was used.

Among the known approaches to modeling strain-induced roughness, two groups can be distinguished. The first group is based on the crystal plasticity (CP) theory [24]. This approach takes into consideration the crystal lattice orientation of the grains. However, there are at least two good reasons why the practical application of such an approach might be ineffective.

1. Significant difficulties during the methodology calibration, especially for hot deformation.
2. Inhomogeneity in the mechanical properties of the material are not only related to differences in the crystal lattice orientation, as was assumed in this approach. Some differences in the microstructure, the presence of non-metallic inclusions, or the second phase of the material can also be important.

The complexity of the CP model and the process of its calibration is such that the construction of such a model goes far beyond the scope of the practical problem solution considered by us in this study.

The second approach uses classical mechanics of continuous media to model inhomogeneity in the mechanical properties of the microstructure element [25]. The finite element method (FEM) was used in this paper to understand strain-induced roughness. Failure to show the method of model calibration and insufficient verification of its results are serious shortcomings of this paper. The model calibration methodology using results of nanoindentation measurements has been proposed by Milenin et al. [26]. It was assumed that the statistics in the dispersion of the nanoindentation results over the cross-section of the material can be transferred to the statistics of the flow stress distribution in grains of the microstructure. The usefulness of this assumption was confirmed for the CDD of magnesium alloy tubes. The same approach and methodology were used in this study for the IDD process.

In this study, the hypothesis that proper selection of IDD process parameters will allow obtaining a cheaper product with better workability and lower surface roughness than the product obtained by the CDD technology will be verified. The practical goal of this study is to propose an inexpensive technology for the production of thin wires (thickness of 140 mm) from the brass CuZn37. This wire is commonly used as electrically charged wire in electric discharge machining [27] and in jewelry manufacturing [28].

To do this, some partial goals have to be achieved:

(i) Develop a theoretical model of the roughness changes during DD and the model calibration based on the experimental results for thin brass wire;
(ii) Comparative analysis of roughness development during the CDD and IDD processes and explanation of the reasons for any possible differences.

## 2. Materials

**Figure 1:**
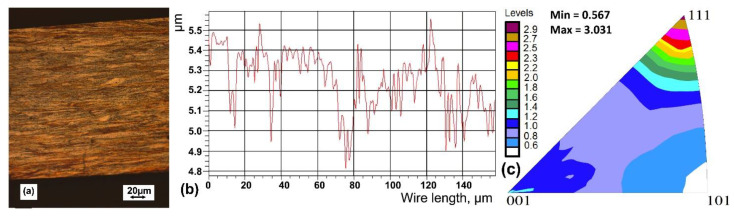
Results of the experimental study of the initial wire: (**a**) microstructure (light microscope); (**b**) example surface profile in the longitudinal direction; (**c**) crystallographic textures of initial wire (the inverse pole figure).

Commercial cold-drawn wire with a diameter of 200 µm made of CuZn37 alloy was used as the DD workpiece. The wire microstructure with grains elongated in the drawing direction (Figure 1a) is typical for cold-drawn materials. The metallographic microscope Axio Imager M1m by ZEISS (Oberkochen, Germany) was used for microstructure characterization. The arithmetic average roughness (Ra) of the surface measured along the wire length was equal to 0.12 ± 0.04 µm (Figure 1b). According to relevant standards (ISO 4288, ISO 21920-3, and ASME B46.1), the sampling length was equal to 0.8 mm, and the evaluation length was 5 times higher (i.e., equal to 4 mm). The optical profiler Wyko NT930 by Veeco (New York, NY, US) was used for the surface roughness measurements.

The Empyrean PANalytical X-ray laboratory diffractometer (by Malvern Panalytical, Malvern, UK) equipped with a parallel Goebel mirror (divergence 0.02°) in the incident beam, two soller slits (0.04 rad) placed on both incident and diffracted beams, and a parallel-plate collimator (divergence 0.18°) was applied in the XRD incomplete pole figures measurements. CuKα radiation was used, and a nickel filter was applied in front of the PIXCel 3D detector. The inverse pole figures were calculated using the LaboTex Software by Labo Soft Company (Kraków, Poland) [29] from the incomplete pole figures 111, 200, 220, and 311, measured by the Schulz reflection method [30]. The measurement covered the angular positions of the sample α  =  0–80° (tilting) and β  =  0–60° (rotation around the normal direction) and were performed in net points Δα  =  Δβ  =  5°. However, only a tilting range up to 65° was used for the complete figure calculation. The [111] fiber texture, typical for drawn wire made of *fcc* structure metal, was revealed (Figure 1c). Some duplex fiber textures of [111] and [100] orientations are usually developed in brass wire conventionally drawn, and the former is usually much stronger. In our wire texture, the component [100] was almost completely absent, which can be associated with a relatively large amount of cold deformation. Such crystallographic texture can promote strain-induced roughness during the DD process.

The stress–strain curves of the CuZn37 alloy were determined from compressive tests made on the Zwick250 machine by ZwickRoell GmbH & Co. (Ulm, Germany). Based on the result, the flow stress dependence on the strain, strain rate, and temperature was determined [31] as:

(2) $$\sigma =Ae^{m_{1}t}\varepsilon^{m_{2}}\xi^{m_{3}}e^{m_{4}/\varepsilon }{\left(1+\varepsilon \right)}^{m_{5}t}e^{m_{6}\varepsilon }\xi^{m_{7}t}t^{m_{8}}$$

where ε—plastic strain, ξ—strain rate, *t*—temperature, *A*, *m*_1_–*m*_8_ are empirical coefficients: *A* = 81259.14073, *m*_1_ = −0.004279279, *m*_2_ = 0.093835212, *m*_3_ = −0.036575464, *m*_4_ = −0.004250721, *m*_5_ = 6.06186·10^−5^, *m*_6_ = −0.4821499, *m*_7_ = 0.000317437, and *m*_8_ = −0.596443559.

**Figure 2:**
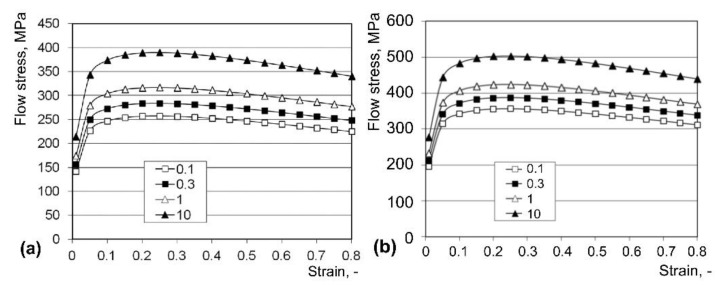
Stress–strain curves of CuZn37 alloy for 400 °C (**a**), 350 °C (**b**), and different strain rates (0.1–10 s^−1^).

Some examples are shown in Figure 2.

## 3. Methods

### 3.1. Overall Research Approach

**Figure 3:**
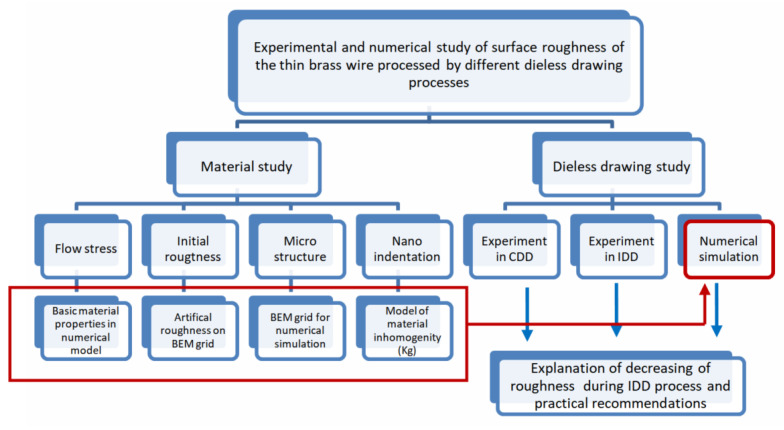
Overall flowchart of the research method.

The flowchart of the research method is shown in Figure 3.

### 3.2. Dieless Drawing Processes

**Figure 4:**
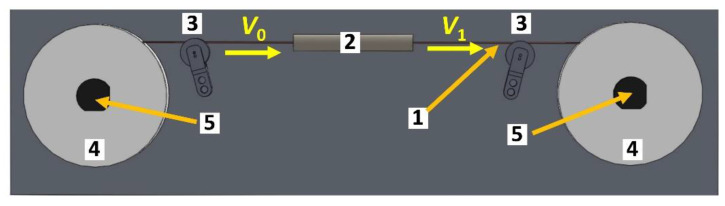
Scheme of the equipment for dieless drawing: 1—wire; 2—heating device; 3—support rollers; 4—work rollers; 5—electric stepper motors.

The schematic of the setup used for DD is shown in Figure 4. Work rollers (4) rotate at different speeds controlled by dedicated PC software, providing the deformed wire velocities *V*_0_ and *V*_1_ (*V*_1_ > *V*_0_). The difference in the velocities was used for the wire longitudinal strain calculation under the constant volume assumption from the formula:

(3) $$\varepsilon_{pass}=1-\frac{V_{0}}{V_{1}}$$

Since there is no friction between the material and the die surface during deformation, the material velocity is theoretically unlimited. However, an increase in the velocity requires an increase in the heating device power, since the material heating time is inversely proportional to the velocity. Practically used material velocities cover the range of 0.25–1.1 m/s [4,32]. The difference between the velocities *V*_0_ and *V*_1_ determines the elongation of the wire and is limited by the workability of the material under the given conditions.

An electric mini-furnace 2 cm long and 0.5 mm in diameter was used as the heating device (2 in Figure 4). The length of the heating device corresponds approximately to the length of the deformation zone.

The length of the processed wire is limited only by the capacity of the work rolls. With a wire diameter of 200 μm, rolls (4 in Figure 4) can contain up to 10–15 m of wire. In our studies, wire samples with a length of 3–5 m were processed without any problems.

Two DD processes were performed on this device, i.e., CDD and IDD. For the one-pass CDD process, the velocity *V*_1_ monotonically increased until final elongation or wire breakage. To measure the dependence of roughness on elongation, the process was repeated several times up to different final elongations.

To carry out the multi-pass IDD process, the rollers’ rotation direction was reversed at precise moments in time. Each pass strain amount was much smaller than in CDD and was carefully selected to ensure the deformation stability. The passes were repeated many times, and surface roughness was measured at a different stage of the process.

### 3.3. Roughness Measurement

For the calculation of the *R_a_* parameter, the following equation was used:

(4) $$R_{a}=\frac{1}{n}\overset{n}{\underset{i=1}{\sum }}\left|y_{i}\right|$$

In this formula, it is assumed that the mean line of the profile has been calculated, where *y_i_* is the vertical distance from the mean line to the data point, and n is the number of ordered points in the profile [33]. Equation (4) was also used for the calculation of roughness in the mathematical model, but instead of the measured values of *y_i_*, coordinates of corresponding nodes in the numerical model were used.

### 3.4. Boundary Element Method Based on the Numerical Model of Roughness

The model developed in this study is based on the boundary element method (BEM) applied to the simulation of the deformation of a representative volume element (RVE) consisting of several tens of model grains. The conditions for the joining of grains are added to the system of equations of the problem. Each grain can have individual mechanical properties. Interpolation of unknown quantities inside grains (displacements and stresses) is based on the fundamental Kelvin solution, transformed for the two-dimensional problem of visco-plastic deformation of an incompressible material [26] expressed by equations:

(5) $$∆u_{x}=\frac{3\;F_{x}}{2E_{g}}\left(g\left(3-4\nu \right)-x\frac{\partial g}{\partial x}\right)-\frac{F_{y}y\left(1+\nu \right)}{E_{g}}\frac{\partial g}{\partial x}$$

(6) $$∆u_{y}=\frac{F_{y}\left(1+\nu \right)}{E_{g}}\left(g\left(3-4\nu \right)-y\frac{\partial g}{\partial y}\right)-\frac{F_{x}x\left(1+\nu \right)}{E_{g}}\frac{\partial g}{\partial y}$$

(7) $$\sigma_{x}=F_{x}\left(2\left(1-\nu \right)\frac{\partial g}{\partial x}-x\frac{\partial^{2}g}{\partial x^{2}}\right)+F_{y}\left(2\nu \frac{\partial g}{\partial y}-y\frac{\partial^{2}g}{\partial x^{2}}\right)$$

(8) $$\sigma_{y}=F_{y}\left(2\left(1-\nu \right)\frac{\partial g}{\partial y}-y\frac{\partial^{2}g}{\partial y^{2}}\right)+F_{x}\left(2\nu \frac{\partial g}{\partial x}-y\frac{\partial^{2}g}{\partial y^{2}}\right)\;$$

(9) $$\sigma_{xy}=F_{x}\left(\left(1-2\nu \right)\frac{\partial g}{\partial y}-x\frac{\partial^{2}g}{\partial x^{2}}\right)-F_{y}\left(\left(1-2\nu \right)\frac{\partial g}{\partial x}-y\frac{\partial^{2}g}{\partial x\partial y}\right)$$

(10) $$g=-\frac{1}{4\pi \left(1-\nu \right)}ln\sqrt{x^{2}+y^{2}}$$

where $\sigma_{x}$*,* $\sigma_{y}$, $\sigma_{xy}$—stresses in nodes on grains boundaries; $F_{x}$, $F_{y}$—forces that result in increments of displacements and stresses in Kelvin′s fundamental solution; $∆u_{x}$, $∆u_{y}$—displacement increments in nodes on grains boundaries; *ν*—Poisson’s ratio (for an incompressible material equal to 0.5).

The algorithm of the model is based on determining the loads $F_{x}$, $F_{y}$ at the nodes of the boundary elements mesh when the boundary conditions are met. Knowing the loads at the nodes, increments in displacement components, and the stresses in nodes can be determined by Equations (5)–(10). A detailed description of the model algorithm is given elsewhere [34,35] (basic principles and their application and the code validation, respectively).

To determine the value of strains increments and strain rates in grains, the central point of each grain must be defined. Some increment in this point displacement was determined as the average of displacement increments in the nodes of the boundary elements from the current grain. Based on the linear interpolation of $∆u_{x}$ and $∆u_{y}$ the increments in each grain strain were determined by Cauchy equations (that follow from the Saint–Venant compatibility condition; for details see, for example, [36]). The following conditions imposed on the boundaries were determined experimentally: longitudinal strain of wire, $\varepsilon_{x}$; strain rate, ξ; and temperature, *t*.

The material of the grains is characterized by the current values of the strain rate, strain, and flow stress following Equation (2). To take into consideration the difference in the mechanical properties of various grains, the *K_g_* coefficient is introduced in the model, the values of which are distributed in the grains under the normal distribution law of random variables. The parameters of this distribution are determined based on processing the results of experiments on nanoindentation. In the Kelvin equations, the mechanical properties of grains are taken into consideration using the effective modulus *E_g_*, which in the model is calculated by the equation:

(11) $$E_{g}=K_{g}\frac{\sigma \left(\varepsilon ,\dot{\varepsilon },t\right)}{∆\varepsilon }$$

where $\sigma \left(\varepsilon ,\dot{\varepsilon },t\right)$ is the flow stress (Equation (2)), ∆ε is the increment of effective plastic strain in grains, and *K_g_* is a coefficient that takes into consideration the inhomogeneity of mechanical properties in different grains.

Each grain value $E_{g}$ is calculated by the iterative method, the values of $\varepsilon ,\dot{\varepsilon }$ are determined as an average over the volume of the grain, and each grain temperature is taken to be the same.

**Figure 5:**
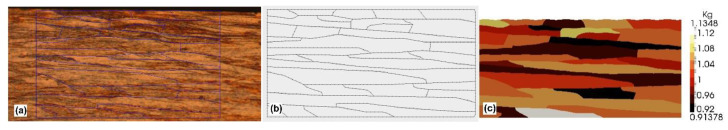
Stages of preparing a numerical model of a representative volume element: (**a**) processing of microstructure image and generation of grain boundaries; (**b**) generated BEM grid; (**c**) generated BEM grid with artificial initial roughness and distribution of the Kg parameter.

The computational grid was built in such a way as to recreate the real grain microstructure observed through an optical microscope (Figure 5a). The image analysis software described elsewhere [37] was used for the grain boundary identification in the microscopic images of the real microstructure and for the generation of the corresponding microstructure of the domain used in the computer model (see Figure 5b). After that, an artificial surface relief was imposed on the calculation mesh (Figure 5c) in accordance with the measured roughness of the initial wire (Figure 1b). In the model, the classical definition of the roughness parameter Ra (i.e., the arithmetic average of the absolute values of the profile height deviations from the mean line, recorded within the evaluation length (EN ISO 4287:2000, ASME B46.1-19950)) was slightly changed. Thus, the surface profile height was referenced to the computational network nodes lying on the model wire surface along its length. Thus, to perform numerical studies, the model needs calibration with the following particular steps:

- The flow stress model determination (Equation (2));
- Generation of the boundary elements (BEM) grid based on experimental data (i.e., microstructure, Figure 5a);
Determination of the mechanical inhomogeneity $K_{g}$ parameter values (Equation (11)), based on the nanoindentation test results;
- Generation of the surface relief in BEM grid based on experimental data (i.e., initial surface profile modeling) (Figure 5c).

### 3.5. Nanoindentation Tests for Calibration of Numerical Model

Nanoindentation tests were performed using the Hysitron Tribolab TI-700 instrument by Bruker Co. (Billerica, MA, US) equipped with the Berkovich tip indenter. Reduced elastic moduli (*E_r_*) were evaluated using the methodology proposed by Oliver and Pharr [38]. Subsequently, statistical analysis and data deconvolution were used to determine single-phase distributions following the methodology proposed by Němeček et al. [39] and Gupta et al. [40]. Each measurement was performed under displacement controlling with a maximum displacement of 200 nm. The trapezoidal load function was applied (i.e., 3.7 s of the linear loading/unloading and 5 s of the holding period). Sixty hardness measurements were made, placed in five rows parallel to the wire diameter. Before the measurement, the wire was placed in a molting form, fixed by adhesive tape to define the grinding plane, and poured into epoxy resin. Then, it was gently ground mechanically using SiC foils up to grit size 4000 with water as a lubricant. This way, approximately half of the wire thickness was removed. Afterward, the wire was polished until a mirror shine was achieved, using the Struers (Copenhagen, Denmark) diamond suspensions with the particle size of 3 and 1 µm. After a thorough cleaning of the surface of the remaining polishing suspension, the sample was ready for measurement.

## 4. Results and Discussion

### 4.1. Results of the Nanoindentation Test and Calibration of the BEM Model

**Figure 6:**
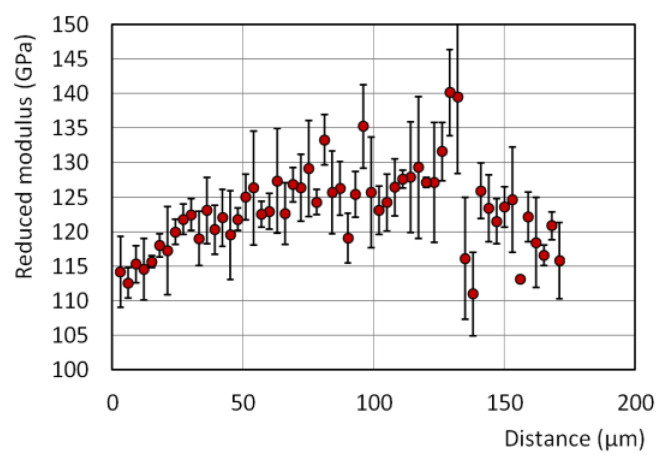
Reduced modulus vs. the distance from the longitudinal symmetry axis of the initial wire: nanoindentation test result.

Results of the nanoindentation test are shown in Figure 6 in the form of the reduced module *E*_r_ dependence on the distance from the longitudinal symmetry axis of the initial wire.

The mean value of the reduced module $\bar{E_{r}}$ and mean standard error $\Delta E_{r}$ calculated for the whole data set was equal to $\bar{E_{r}}$ = 123.2 GPa and $\Delta E_{r}$ = 4.416 GPa, respectively. These values were used to determine the *K_g_* parameter value by the following equation:

(12) $$K_{g}=1\pm \frac{∆E_{r}}{\bar{E_{r}}}$$

A *K_g_* value equal to 1 ± 0.0493 was used as the basic data of an artificial generator of random variables $K_{g,\;i}$ for *i*-th grains of the model microstructure distributed according to the normal distribution (Figure 5c). For this purpose, the *vdrnggaussian* function included in the Intel^®^ (Santa Clara, CA, US) Math Kernel Library, version 2021.1 was used in the dedicated software developed by us.

### 4.2. Experimental Study of the DD and IDD Processes

**Figure 7:**
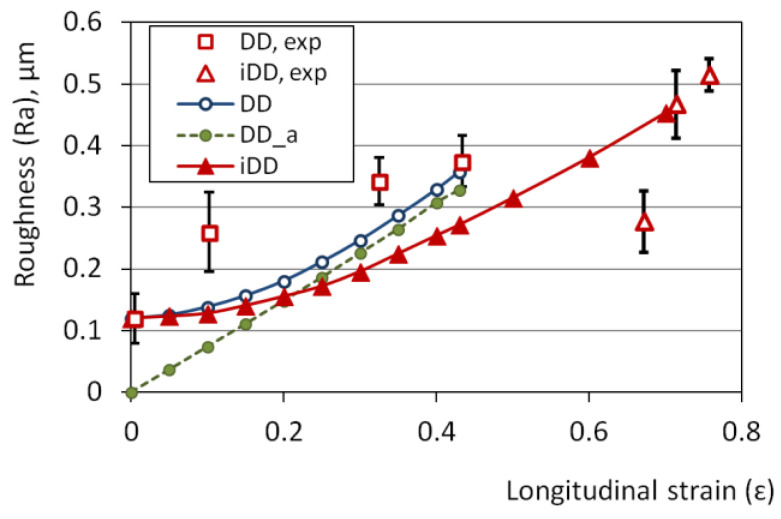
Results of experimental and numerical study of the strain effect on roughening for incremental dieless drawing (IDD) and conventional dieless drawing (CDD). In the figure, calculated and experimental (exp.) data for CDD and IDD are shown. The initial roughness Ra was equal to 0.12 µm (IDD and CDD) and 0 µm (CDD (a)).

The experimental conditions of the drawing processes are collected in Table 1. Each of the DD processes listed in the table were repeated several times for different final deformations of the wire. The longitudinal strain was calculated from the results of the measurement of the change in the wire diameter. The maximum achieved strain was equal to 0.43 and 0.78 for the CDD and IDD processes, respectively (increasing in the workability by more than 1.8 times). The roughness versus strain relationship is shown in Figure 7.

### 4.3. Strain Effect on Roughness Modeling

**Figure 8:**
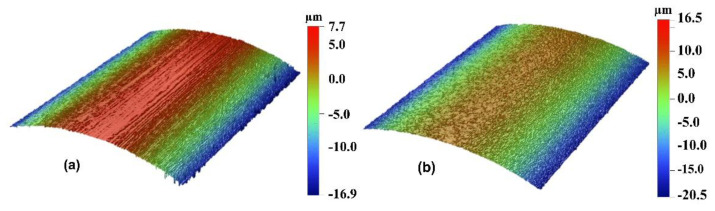
Typical three-dimensional surface profile of wire: (**a**) initial wire; (**b**) wire after IDD process.

Experimental studies have shown that both processes (i.e., CDD and IDD) significantly change the surface profile (e.g., Figure 8). So, longitudinal grooves (i.e., traces of the wire drawing process) visible on the original wire are covered by the surface profile developed in the IDD/CDD process. Qualitatively, the surface profile developed by the CDD and IDD processes is very similar. The quantitative difference in surface roughness development during both of the above processes were determined by the numerical modeling.

The plastic strain effect on surface roughness was numerically simulated for IDD and CDD (for both, the initial roughness *R_a_* was equal to 0.12 µm) and additionally for CDD for an initial *R_a_* equal to 0 µm (CDD_a). Identical grain microstructure (Figure 5a,b), computational networks, and the *K_g_* parameter distribution (Figure 5c) were used for each of the above cases. The flow stress in grains was calculated with Equation (2), and the simulated experimental conditions were collected, as shown in Table 1.

**Figure 9:**
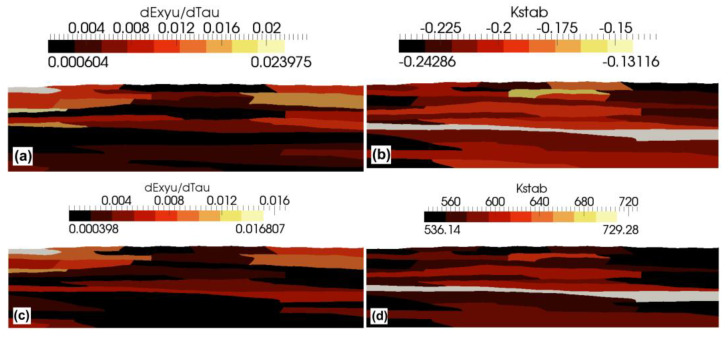
Numerical simulation results were obtained for the CDD process (**a**,**b**) and IDD process (**c**,**d**); distribution of the shear strain rate in s^−1^ (**a**,**b**) and the coefficient of the plastic deformation stability (**b**,**d**).

For a perfectly smooth surface without any roughness (i.e., the initial value of *R_a_* equal to 0 µm), the dependence of the parameter *R_a_* calculated from the model on the wire deformation in CDD can be well described by a linear function of the form: *R_a_* = 0.7574*ε* (the coefficient of determination R^2^ equal to 0.9997), where *ε* is the longitudinal strain of the wire. Some initial roughness reduces the intensity of its growth upon the CDD strain for a low deformation range (up to strain of about 0.3 in the case shown in Figure 7). Above, a similar growth rate in *R_a_* value as for the perfectly smooth wire was revealed. The model results showed that the influence of the initial roughness on the roughness of a strongly deformed wire is relatively small. As the deformation of the CDD wire increases, the results obtained from the model become closer to the experimental results (Figure 7). A much slower growth rate in the *R_a_* value over the entire strain range was model predicted for the IDD with a strain increment of 0.09, and approximately a 30% lower *R_a_* was predicted by the model for the limit strain in CDD, equal to 0.43. This effect seems to be related to lover mean shear strains and to higher stability of the grain deformation (i.e., higher value of the *K_stab,i_* parameter) predicted by the model for IDD than for CDD (see Figure 9). Higher deformation stability means that stress rises faster in the grains deforming. This, as a result, leads to more homogeneous deformation, since the local increase in the strain is immediately impeded by the strain hardening. It can be summarized that more homogeneous deformation of the grains reduces strain-induced roughness, which can be observed in Figure 7.

## 5. Summary

The roughness of CuZn37 wire strain induced during dieless drawing was explained based on experimental and numerically predicted results obtained for conventional and incremental dieless drawing. A small increase in strain (about 0.09) was imposed on the latter of these processes. Approximately a 30% reduction in roughness was experimentally demonstrated for the incremental dieless drawing process carried out under the same conditions as the conventional process. Numerical microscale modeling showed that a decrease in strain-induced roughness occurs due to the more stable plastic deformation of the grains connected with the incremental dieless drawing process.

A decrease in the roughness of the wire surface during incremental dieless drawing is also accompanied by an increase in the workability by more than 1.8 times. The incremental dieless drawing process, however, has lower productivity due to the need to perform multiple passes instead of just one in the conventional process.

## Figures and Tables

**Table 1:** The experimental conditions of the DD processes.

Process	*V*_0_, mm/s	*V*_1_, mm/s	*ε*_pass_	*n*	*t_d_*, °C
CDD	12.16	12.16–18.1	0–0.328	1	400
IDD	12.16	13.38	0.091	8	400

*V*_0_, *V*_1_—wire velocities; *ε*_pass_—longitudinal strain (according to Equation (3)), *n*—number of passes, *t_d_*—the temperature in a heating device, measured by a thermocouple.

## Data Availability

Data are contained with the article.
